# Realistic wave-optics simulation of X-ray phase-contrast imaging at a human scale

**DOI:** 10.1038/srep12011

**Published:** 2015-07-14

**Authors:** Yongjin Sung, W. Paul Segars, Adam Pan, Masami Ando, Colin J. R. Sheppard, Rajiv Gupta

**Affiliations:** 1 Department of Radiology, Massachusetts General Hospital and Harvard Medical School, Boston, Massachusetts 02114, USA; 2 Department of Radiology, Duke University, Durham, North Carolina 27705, USA; 3 Harvard-MIT Division of Health Sciences and Technology, Cambridge, Massachusetts 02139, USA; 4 The Research Institute for Science and Technology, Tokyo University of Science, Noda, Chiba 278-8510, Japan; 5 Istituto Italiano di Tecnologia, via Morego 30, Genova 16163, Italy

## Abstract

X-ray phase-contrast imaging (XPCI) can dramatically improve soft tissue contrast in X-ray medical imaging. Despite worldwide efforts to develop novel XPCI systems, a numerical framework to rigorously predict the performance of a clinical XPCI system at a human scale is not yet available. We have developed such a tool by combining a numerical anthropomorphic phantom defined with non-uniform rational B-splines (NURBS) and a wave optics-based simulator that can accurately capture the phase-contrast signal from a human-scaled numerical phantom. Using a synchrotron-based, high-performance XPCI system, we provide qualitative comparison between simulated and experimental images. Our tool can be used to simulate the performance of XPCI on various disease entities and compare proposed XPCI systems in an unbiased manner.

The image contrast of conventional X-ray imaging primarily arises from the attenuation of X-rays due to photoelectric absorption and Compton scattering^1^. This attenuation contrast is sensitive to differences in atomic number. Thus, bones are distinctly differentiated from soft tissues in X-ray radiography, but structures within soft tissues are difficult to detect. X-ray phase-contrast imaging (XPCI) utilizes an alternative contrast mechanism to generate images, namely, phase alteration or refraction of X-rays due to electron clouds of various materials^2^. As refraction is sensitive to sharp edges, the phase-contrast signal from soft tissues can be as strong as the attenuation-contrast signal from bones^2^. As a result, XPCI can, for example, characterize soft-tissue components of atherosclerotic plaque and discriminate benign from cancerous tissues^3^,^4^,^5^,^6^. The angular deviation in typical X-ray refraction is quite small, on the order of a μrad. A variety of XPCI methods have been proposed to capture this small signal first using synchrotron radiation^7^,^8^,^9^,^10^,^11^ and then on the laboratory scale^12^,^13^,^14^,^15^,^16^,^17^. Extensive efforts are now being made to increase the imaging volume and translate this technology to a clinical scale^18^,^19^.

Simulation serves as an essential tool for characterizing, evaluating, and optimizing new imaging technologies; it provides realistic predictions of images without complications due to uncontrolled experimental parameters. Using a model of the imaging process, images can be acquired from a phantom of human anatomy as if it were a live patient; therefore, it is possible to perform clinical experiments entirely *in silico*. The advantage in using such studies is that the exact anatomy of the phantom is known, and provides a “gold standard” or “ground truth” from which to quantitatively evaluate and improve imaging devices and techniques. Simulating XPCI at a human scale poses great challenges in both numerical phantom and imaging model development. Voxelized phantoms, typically adopted in medical imaging simulation, cannot be used for XPCI simulation because the boundaries of discretized meshes generate strong artifacts in XPCI simulation. For image formation, existing methods such as ray tracing^20^,^21^ and Monte Carlo^22^ methods are unsuitable because they do not account for the wave nature of X-rays. For example, these methods do not model coherent scattering and diffraction within the patient, processes that are responsible for imparting the phase signal. Accurate modeling of these interactions becomes more difficult as the size of numerical phantom increases. As a result, there is currently no human-scaled XPCI simulator, which makes it difficult to predict the performance of various XPCI systems and compare their advantages and disadvantages in a clinical scenario.

We have developed a simulation framework that can generate realistic pictures of phase-contrast imaging at an adult human scale. The 4-D extended cardiac-torso (XCAT) phantom represents complex human anatomy using non-uniform rational B-splines (NURBS). Our wave optics-based simulator can accurately predict the propagation of X-ray ‘waves’ through the NURBS-defined organs. Using the developed tool, we generate, for the first time to the best of our knowledge, projection images of a human thorax with both attenuation and phase contrast. The simulated images clearly show the superiority of XPCI over conventional attenuation-based X-ray imaging for visualizing various soft-tissue structures. Currently, there is no XPCI system that can image a human torso. Using X-ray dark-field imaging (a synchrotron-based XPCI modality), we provide qualitative comparison of simulated human chest images with experimentally acquired frog images.

##  

### X-ray phase-contrast imaging simulation with NURBS-defined human phantom

Maxwell’s equations describe the propagation of electromagnetic waves, including X-rays, through a general medium^23^. Maxwell’s equations can be reduced to a scalar wave equation when the medium is a linear dielectric and its properties are independent of the polarization direction of the incident wave^23^. The wave equation can be solved numerically, but it requires iterative computation on a volume mesh or a 3-D grid that covers a large computational domain^24^. Importantly, to capture the phase alteration in X-rays caused by interaction with a human subject, a sub-nanometer grid must cover a computational domain ten-orders-of-magnitude larger than the grid size. Storing data on such a 3-D grid is almost impossible, letting alone solving the wave equation on it. Therefore, simplification of the wave equation is typically introduced for computational efficiency using the Born or the Rytov approximation. The Born approximation is valid when the optical thickness of the object is smaller than the wavelength, while the Rytov approximation is valid as long as the refractive index gradually changes over the wavelength scale within the imaged object and at the boundaries^25^. Therefore, the Rytov approximation can be safely used for XPCI simulation, whereas the Born approximation cannot. The Born and Rytov solutions have been adopted in phase imaging or phase retrieval problems to describe the free-space propagation after the object^26^,^27^. In our previous work^28^, we used the Rytov solution to describe the X-ray-object interaction as well as the free-space propagation, and confirmed its validity using the Mie solution, the exact solution of Maxwell’s equations.

The Rytov solution calculates the amplitude and phase of X-rays on the detector directly from the object’s 3-D scattering potential. Figure 1 schematically shows this calculation process, using a coronary artery model. Suppose that parallel X-rays are incident on the object and the scattered X-rays are recorded at a certain distance from the object. The scattering potential, which forms a key input to the simulation, represents both geometric and material information of the object (Fig. 1a). For a specific energy and direction of the incident beam, the recorded image contains the object information that corresponds to the spatial-frequency components lying on the Ewald sphere^23^. In digital imaging using discretized pixels, this corresponds to sampling the object’s spectrum at the grid points defined by the Ewald sphere and projecting the sampled values onto the 2-D spatial frequency coordinates of detector (Fig. 1b). The projected spectrum is multiplied with a scale factor that represents free-space propagation (Eq. (3)). The result of this operation is called the angular spectrum (Fig. 1c), whose 2-D inverse Fourier transformation can be connected to the amplitude and phase of X-ray field on the detector (Eq. (2)). Combining the amplitude and phase images with system-specific image formation and noise models, we can simulate virtually all XPCI methods. For example, Figs 1d,e simulate the coronary artery images (Fig. 1a) acquired in in-line holography XPCI for object-to-detector distances of 1 m and 5 m, respectively. For this simulation, we assumed that parallel X-rays at 30 keV were incident onto the object model. Figure 1F compares cross-sectional profiles along the dotted lines in Figs 1d,e; as can be clearly seen, the edge signal is amplified as the object-to-detector distance increases. This edge enhancement occurs because the object-induced phase variation is encoded into amplitude variation as the light propagates in free space^8^.

The scattering potential of an object is a key input to the XPCI simulation process. In the simplest approach, we can define the object’s scattering potential using a discretized volume mesh with the voxel values obtained from dual-energy CT^29^ or material decomposition^30^. This approach, however, is problematic in two respects. First, the Rytov solution requires sampling the Fourier transform values at the exact grid points defined by the Ewald sphere and the spatial-frequency coordinates of detector (Fig. 1b). Given a discretized volume mesh, this means resampling the discrete Fourier transform of the object at the grid points by interpolation. It is well known that this interpolation in the spatial frequency space generates severe numerical artifacts in the spatial domain^31^. Second, discretized boundaries of a volume mesh generate strong edge signals that can easily overwhelm the phase-contrast signals from real features. For example, Fig. 2a shows a Shepp-Logan phantom^32^ represented by 256 × 256 × 256 voxels (the MATLAB code is available at http://www.mathworks.com/matlabcentral/fileexchange/9416-3d-shepp-logan-phantom). The phantom’s discretized boundaries can be clearly seen in Fig. 2b^33^. Using this type of discretized phantom for XPCI simulation, we observe several ring patterns in the simulated image (Fig. 2c), which are numerical artifacts. Smoothing the phantom with a Gaussian filter helps to reduce the artifact (Fig. 2d), but it also smoothes out phase-contrast signals from small features. Refining a volume mesh quickly increases the computational cost, and cannot provide the accuracy that XPCI requires. The 4-D extended cardiac-torso (XCAT) phantom provides a solution to this problem of discretized representation^34^. The XCAT phantom represents human organs using approximately 2800 non-uniform rational B-spline (NURBS) surfaces that can efficiently and seamlessly represent complex, arbitrary shapes. NURBS has been adopted in medical imaging simulation to improve the accuracy of ray tracing^35^, or to generate volume meshes with arbitrary resolution^36^. An interesting approach to utilizing NURBS has been recently reported, in which the van der Waals force, originating from non-covalent interaction of two neighboring volumes, was calculated using a surface integral over the NURBS surfaces representing the volumes^37^. We adopt this approach to calculate the 3-D Fourier transform of a NURBS-defined object or scattering potential. A complex arbitrary object can be represented by a superposition of homogeneous volumes. The 3-D Fourier transform of a homogeneous volume can be interpreted as the volume integral of a spatial harmonic function 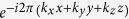 over the volume^38^. Here, (*x,y,z*) are the space coordinates where the object is defined, and (*k*_*x*_*,k*_*y,*_*k*_*z*_) are the spatial frequency coordinates of a point, for which the Fourier transform is calculated. Utilizing the divergence theorem^39^, this volume integral can be substituted by a surface integral of a specially-chosen vector field (See Methods) over the surface represented by NURBS^39^. Therefore, the 3-D Fourier transform of a complex object can be calculated by summing up the surface integrals over the NURBS surfaces representing the object. This approach is computationally efficient compared to directly evaluating the volume integral. The amplitude and phase of X-rays at the detector plane can be simply obtained from the Fourier transform values following the process described above.

### Simulated attenuation- and phase-contrast thoracic radiography

Using the method described in the previous section, we simulate attenuation- and phase-contrast X-ray imaging applied to a human chest phantom. Specifically, we extract about 600 NURBS models representing all the intra-thoracic organs from the XCAT phantom, and assign complex refractive index values to them. These values can be calculated from the elemental composition of tissues^40^ and the physical properties (atomic number, atomic weight, mass attenuation coefficient) of elements at each simulated X-ray energy^1^. For each NURBS model, we calculate both the amplitude and phase of electric field at the detector plane. The electric field for the entire chest is their coherent sum. The attenuation- and phase-contrast images in Figs 3a,b, respectively, are the squared amplitude and the phase angle of the total electric field calculated at 1 m from the center of the phantom. For this simulation, we assumed collimated X-rays at 70 keV and detector pixel resolution 0.125 mm. Noteworthy, the image in Fig. 3A includes the phase-induced amplitude variation due to free-space propagation; thus, it simulates the image acquired in inline holography XPCI for the specific object-to-detector distance (1 m). On the other hand, Fig. 3b is a pure phase image such as the one that an interferometry-based XPCI system would provide. In practice, the coherence length of hard X-rays is so small (tens of nanometers for synchrotron radiation) that interferometry cannot be applied to a thick specimen.

Using the amplitude (Fig. 3a) and phase image (Fig. 3b), we can simulate other XPCI methods. For example, the spatial gradient of phase can be associated with the cumulative refraction angle of X-rays after passing through the object^2^. Thus, the image recorded with a grating-based XPCI system^11^,^12^ can be obtained by taking the gradient of the calculated phase (e.g., Fig. 3b), and multiplying it with the calculated amplitude (Fig. 3a) to account for attenuation of each ray. The images in Figs 4a,b correspond to grating-based XPCI using vertical and horizontal grids, respectively. In crystal-based XPCI, the angular deviation of X-rays by the object is recorded with a crystal, whose transmittance^10^ or reflectance^9^ changes with the incident angle. Scaling the phase gradient using the rocking curve for a crystal, we can also simulate the images acquired in a crystal-based XPCI system. The rocking curve is roughly bell-shaped (Pearson Type VII, more accurately^41^). Assuming that a linear portion of the rocking curve is used, the images in Fig. 4 can also be interpreted as simulating crystal-based XPCI. In the attenuation-contrast image (Fig. 3a), bony structures such as ribs and spines are well seen because of their high attenuation. In addition, low-density structures e.g., air-containing trachea, are also visualized. However, different soft-tissue structures are difficult to distinguish from each other because of their similar attenuation. There is higher contrast between these structures on the phase-contrast images (Figs 4a,b). For example, all the structures obscured by the diaphragmatic silhouette are much better appreciated in the phase images. Similarly, the intra-vertebral disks are seen to better advantage in phase-contrast than in attenuation image. The phase-contrast image also demonstrates the bronchial tree (including the primary, secondary, and tertiary branches) better than the attenuation image. The tertiary structure of bronchial trees is about the same size as the pixel resolution^42^; therefore, its effect on X-ray attenuation (Fig. 3a) or phase alteration (Fig. 3b) is negligible. However, crystal-based or grating-based XPCI records the spatial gradient of phase alteration; therefore, the tertiary structure can be clearly seen in the phase-contrast images (Fig. 4). Sub-organ structures such as intrapulmonary lobules and tissue textures such as muscle layers are not included in the current version of the XCAT model. They are therefore not visualized by either the attenuation or phase-contrast simulated images.

There is currently no XPCI system that can image a human torso; thus, direct comparison of simulation and experiment at a human scale is not possible. Instead, we imaged a frog using a high-performance XPCI method called X-ray dark-field imaging (XDFI) and compared the image with simulated human chest images. XDFI is a synchrotron-based XPCI system using an analyzer crystal in transmission for phase detection (Fig. 5a)^6^. Close to the Bragg angle, the crystal’s transmittance sharply changes with the incidence angle of the X-ray beam onto the crystal. XDFI can thereby record sample-induced angular deviation of X-rays with high spatial resolution (<4 μm) and angular selectivity (<5 μrad). Furthermore, X-rays incident onto the specimen in the XDFI set-up are quasi-parallel (angular divergence <0.3 μrad), and quasi-monochromatic (energy bandwidth < 0.02% of the mean energy). Therefore, we can validate our numerical framework without ambiguity due to the characteristics of a laboratory source. Figures 5b,c show the attenuation- and phase-contrast images, respectively, of a live frog, recorded on our XDFI setup. As consistent with our simulated attenuation image (Fig. 3a), the skeletal system of the frog is shown with high image contrast in the attenuation-contrast image (Fig. 5b). The respiratory system including the trachea, the main stem bronchi, and the lung are better demonstrated on the phase-contrast images than on the attenuation image, which is also consistent with the simulated phase-contrast images (Fig. 4). In Fig. 5c, other soft-tissue structures such as the dorsolateral folds, latissimus dorsi muscle, and skin folds are also seen with much higher contrast in the phase-contrast image.

## Discussion

Existing methods for X-ray imaging simulation rely on ray tracing or Monte-Carlo simulation. Ray tracing is appropriate for calculating the attenuation of X-rays along a line-of-sight, while Monte-Carlo simulation is particularly appropriate for calculating Compton scattering and organ-level radiation dose. Simulating X-ray phase-contrast imaging (XPCI), on the other hand, requires calculating the phase alteration of X-rays, which can be best described by wave optics. Early models for XPCI simulation extended the ray tracing approach to include the wave nature of X-rays. For example, in their pioneering work, Pogany *et al.*^43^ used the Fresnel-Kirchhoff integral together with a 2-D transmittance function obtained under the projection approximation. The projection approximation corresponds to using ray tracing with only the rays parallel to the optical axis, which is safe only when the object is thin and the detector is located far from the object^44^. When the object is thick and consists of many small features, which is typical in medical imaging, the projection approximation cannot be used. Other existing approaches^45^,^46^ similarly ignored coherent scattering and diffraction of X-rays within the object, which are essential contributors to the phase-contrast signals. In this study, we used wave optics to describe the entire process of X-ray ‘wave propagation’ from the source to the detector through various structures of the numerical phantom. We can thereby capture coherent scattering and diffraction from small anatomical features within a large body without loss or distortion. Furthermore, using NURBS to represent the complex human anatomy, our method does not suffer from the artifacts caused by discretized mesh and aliasing.

**Figure 3:**
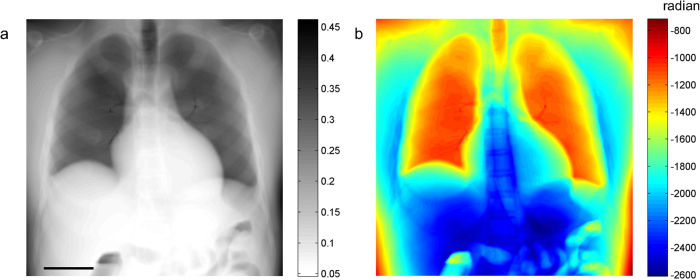
Simulated chest image: (**a**) attenuation; and (**b**) phase alteration measured at 1 m from the numerical phantom. Scale bar, 5 cm.

XPCI methods can be roughly divided into three groups, depending on whether they image the phase, the phase gradient, or the Laplacian of phase^2^. For example, interferometry records the wrapped phase after the object, while grating-based and crystal-based methods record the refraction angle or the phase gradient. Inline holography methods, also known as propagation-based methods, record the Laplacian of phase. There is a misconception that the phase-contrast signal is stronger than the attenuation-contrast signal because the real part of refractive index is typically 1000 times stronger than the imaginary part. However, a fair comparison between the attenuation- and phase-contrast signals has to be made in the image domain. The similarity between the images in Figs 3a,b suggest that the phase contrast is not particularly stronger than the absorption contrast. However, the XPCI methods recording the gradient or the Laplacian of phase amplify the signal from small features. Therefore, the phase-contrast signal from small features such as bronchial trees can be stronger than the corresponding attenuation-contrast signal. Grating-based methods record the gradient of sample-induced phase alteration and can be built on a laboratory source with low spatial and temporal coherence. However, its signal-to-noise ratio is known to be quite low^47^. In-line holography methods can also be built on a laboratory source and provide high signal-to-noise ratio, but require a source with high spatial coherence and a detector with small pixels and high dynamic range^48^. Importantly, new XPCI systems are being developed to overcome the limitations of existing systems^19^. The numerical framework developed in this study can be used to predict the performance of these new systems at a human scale, optimize design parameters (e.g., source voltage, spectrum), and rigorously assess the performance of a reconstruction algorithm.

**Figure 4:**
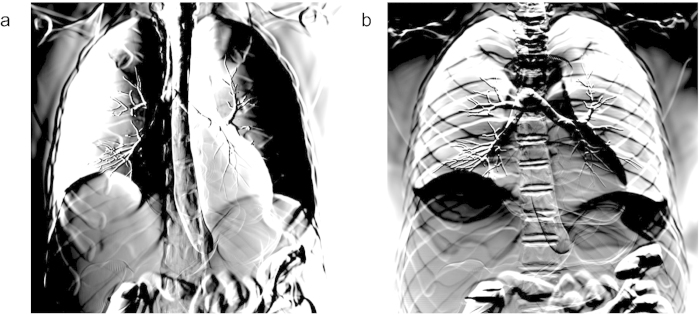
Simulation of an XPCI technique sensitive to the horizontal (**a**) and vertical (**b**) gradients of sample-induced phase alteration.

**Figure 5:**
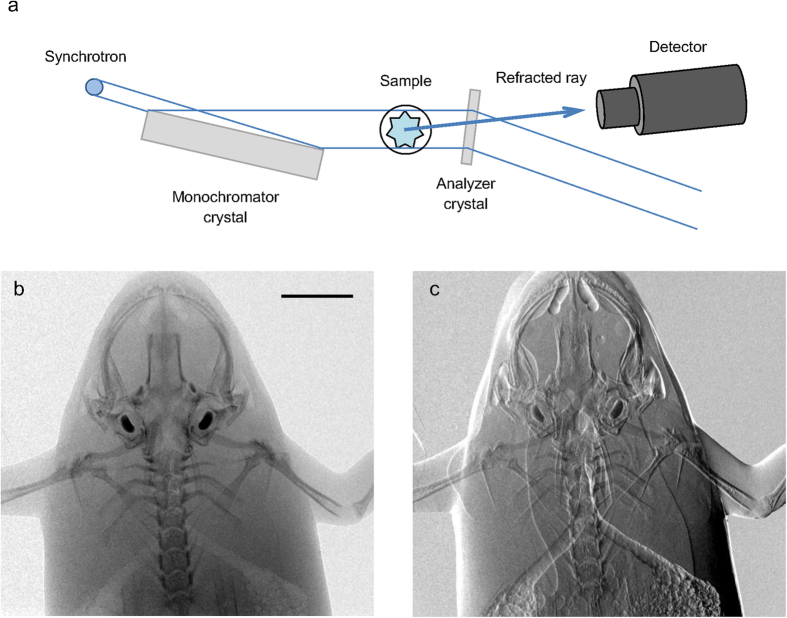
X-ray dark field imaging of a live frog: (**a**) schematic diagram of the set-up used for this study (**a**) and frog images acquired with the set-up: (**b**) attenuation; and (**c**) phase-contrast image (horizontal gradient). Scale bar, 5 mm.

The method described in this study can be extended to a general source with a large focal spot (i.e., arbitrary wavefronts) and a broad energy spectrum using the methodology described in^33^. We simplified the wave equation using the first-order Rytov approximation, whose validity was confirmed using the Mie solution for a homogeneous sphere^28^. A topic for future research is to extend our method to include higher-order interaction or multiple scattering within the object^49^. While the XCAT captures all the macro-structures within the body, it currently lacks sub-structures that form the parenchyma of organs. For example, the micro-level organization of the alveoli within the secondary pulmonary lobules is absent, which explains the discrepancy between the lungs in the simulated human images (Figs 4a,b) and in the frog image (Fig. 5c). Developing a more sophisticated NURBS phantom with detailed sub-organ structures^50^ will be an important research topic for hyper-realistic XPCI simulation.

## Methods

### X-ray wave propagation through a human-scaled object

The scalar wave theory describes the amplitude and phase alteration of electromagnetic waves after interaction with an object^23^:
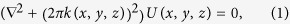
where *λ* is the wavelength of X-rays in vacuum, *n*(*x,y,z*) is the complex refractive index of the object, and *k*(*x,y,z*) = *n*(*x,y,z*)/*λ* is the wavenumber in the object. The wave equation can be solved for the unknown variable *U*(*x,y,z*), the complex amplitude of the wave. Suppose that a monochromatic, collimated X-ray beam is incident on the object. The complex amplitude *U*(*x,y,z*) can be written in the following form without loss of generality^25^,^28^:
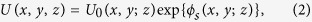
where *U*_0_(*x,y;z*) is the complex amplitude of the incident X-rays, and *ϕ*_*s*_(*x,y;z*) is the complex scattered phase, both measured at a distance *z* from the object. Solving the wave equation with the Rytov approximation ( 
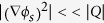
), *ϕ*_*s*_(*x,y;z*) in Eq. (2) can be simply related to the object’s scattering potential *Q*(*x,y,z*) in the spatial frequency space^25^,^28^:
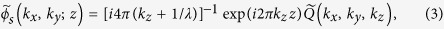
where *Q*(*x,y,z*) = (2*π*/*λ*)^2^(1 − *n*(*x,y,z*)^2^). 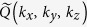 is the 3-D Fourier transform of *Q*(*x,y,z*), and 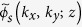 is the 2-D Fourier transform of *ϕ*_*s*_(*x,y;z*) with respect to *x* and *y*. Importantly, *k*_*x*_ and *k*_*y*_ are the spatial frequencies corresponding to the spatial coordinates *x* and *y* in the detector plane, while *k*_*z*_ is given by the following formula for the Ewald sphere^51^: 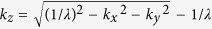.

### The 3-D Fourier transform of the object’s scattering potential represented by non-uniform rational B-splines (NURBS)

Suppose that the 3-D scattering potential of the object can be expressed as a superposition of homogeneous volumes:
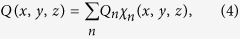
where *χ*_*n*_(*x,y,z*) is the 3-D characteristic function with a value of unity inside the *n*th-volume *V*_*n*_ and zero outside. Taking the 3-D Fourier transform of Eq. (4) expresses 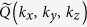 in Eq. (3) as a weighted sum of the 3-D Fourier transforms of the characteristic functions. It is noteworthy that the 3-D Fourier transform of each characteristic function can be thought of as the integral of a function 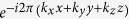 over the bounding volume:
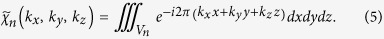


Using the divergence theorem with a proper choice of the vector field 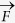, the volume integral in Eq. (5) can be converted to a surface integral^39^:
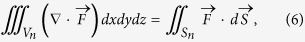


By conjecture, we select the following vector field 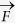, which is continuously-differentiable in space and satisfies the condition 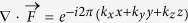:
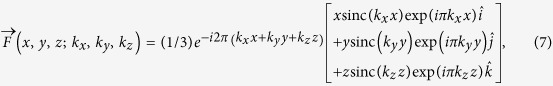
where sinc(*t*) = sin(*πt*)/(*πt*). Suppose that the surfaces defining the object can be parametrically represented using NURBS. A NURBS surface 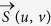 of degree *p* in both the *u* and *v* directions can be defined with a set of 2-D control points 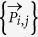 , where *i,j* = 0,1,…,*m*, and two knot vectors {0,…,0,*u*_*p*+1_,…*u*_*m*−*p*−1_,1,…,1} and {0,…,0,*v*_*p*+1_,…*v*_*m*−*p*−1_,1,…,1}^52^:
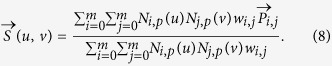


Here, {*N*_*i,p*_(*u*)}and {*N*_*i,p*_(*v*)} are the basis functions for NURBS. Summing up the results from Eqs. (6) through (8), the right hand side of Eq. (6) can be obtained from
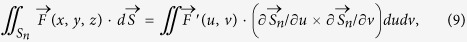
where 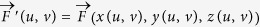. The vector field 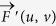 and surface normal vectors 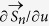 and 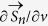 are evaluated on the 2-D knot vectors (*u*,*v*) defining the NURBS surface. The integral on the right hand side of Eq. (9) can be numerically evaluated using Simpson’s 3/8 rule, which is based on a cubic interpolation of the integrand function^53^. The accuracy of the numerical integral can be improved by refining the grid size for the control points. The spatial coordinates and surface normal vectors at each grid point (*u*,*v*) of the 2-D knot vector can be obtained using the algorithm described in Piegl and Tiller^52^. This algorithm was implemented in a MATLAB (Mathworks, Inc.) code by D. M. Spink (http://www.aria.uklinux.net/nurbs.php3).

### Complex refractive index value of a compound material

In the X-ray regime, an object can be represented by complex refractive index 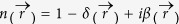
2. For X-rays of wavelength *λ*, the complex refractive index can be calculated from the electron density *N*_*el*_ and linear attenuation coefficient *μ*(*λ*) for the material^2^.
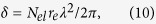

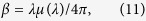
where *r*_*e*_ is the classical radius of electron (2.818 × 10^−15^ m). The mass attenuation coefficient (*μ*/*ρ*)_*c*_ for a compound is simply a weighted sum of the mass attenuation coefficients for the constituent species^30^.
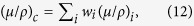
where *w*_*i*_ and (*μ*/*ρ*)_*i*_ are the mass fraction and the mass attenuation coefficient for *i*-th species.

The electron density of a compound material can be calculated from the following^30^.
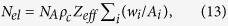
where *N*_*A*_ is the Avogadro’s number, and *ρ*_*c*_ and *Z*_*eff*_ are the mass density and the effective atomic number, respectively, of the compound. The variables *A*_*i*_ and *Z*_*i*_ represent the atomic weight and atomic number of *i*-th species, respectively. The effective atomic number *Z*_*eff*_ of a compound material can be calculated from 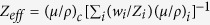
30.

### Parallel computation using graphics processing unit

Simulations are performed on four NVIDIA Tesla C2050 GPUs, and projections typically take about two weeks to complete the computation for all the chest models. The NURBS model is first loaded into all GPU workers. Each GPU is assigned a section of the final image, and applies a parallel transform-reduce operation onto the NURBS model corresponding to the forward model onto each pixel. Compared to voxel representations, NURBS models are very memory efficient, and even highly detailed models only consume ~100 s of megabytes of memory. Instead of accessing the NURBS model each time for calculating each pixel value at the detector, we calculate multiple pixel values in a grouped process within the GPU, reducing the number of reads from memory.

### X-ray dark-field imaging of a frog

X-ray dark field imaging (XDFI) is a synchrotron-based XPCI technique adopting an analyzer crystal in transmission mode^6^,^10^. The XDFI setup (Fig. 5a) comprises of a monochromator crystal, an analyzer crystal, and a CCD camera. For the X-ray source, we use a synchrotron at the beamline BL14C in the Photon Factory (KEK, Tsukuba, Japan). We adopt a thin (Si (440), 520 μm-thick) crystal in transmission Laue mode, which provides high spatial resolution compared to reflection-mode operation. Importantly, we cut a monochromator crystal (Si (440), Bragg-type) such that the cutting plane is at an angle to the crystal plane (tilting angle, 10.2°). This asymmetrically-cut crystal expands the beam at 35 keV by a factor of 50, allowing full-field imaging of a large sample without scanning. To record the images, we use an X-ray detector (X-FDI, Photonic Science Ltd.) with pixel size of 7.4 μm, exposure time of 55 seconds, and the field of view of 36.1 (horizontal) × 24.0 (vertical) mm^2^. The frog experiment was performed in accordance with the guidelines (No. 2008S2-002, 2011G-672, and 2012G558) approved by the PF Program Advisory Committee at KEK.

## Additional Information

**How to cite this article**: Sung, Y. *et al.* Realistic wave-optics simulation of X-ray phase-contrast imaging at a human scale. *Sci. Rep.* **5**, 12011; doi: 10.1038/srep12011 (2015).

## Figures and Tables

**Figure 1:**
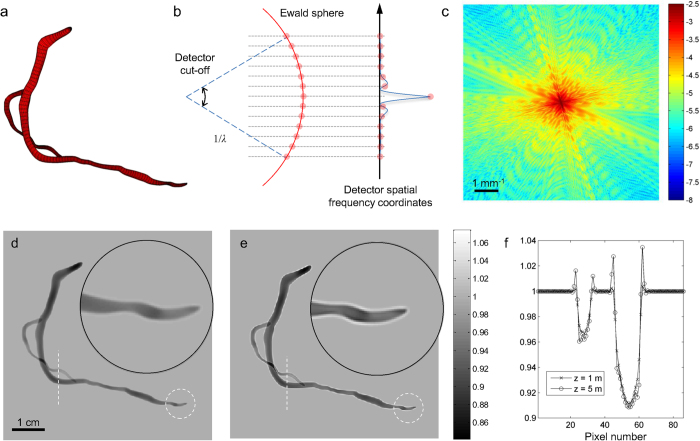
Schematic diagram of image generation in the proposed method. (**a**) A coronary artery model; (**b**) sampling the Fourier transform of (**a**) at the grid points on the Ewald sphere; (**c**) 2-D projection of (**c**). (**d**) and (**e**) show simulated intensity images at z = 1 m (**d**) and 5 m (**e**) from the model. (**f**) shows cross-sectional profiles along the dotted lines in (**d**) and (**e**).

**Figure 2:**
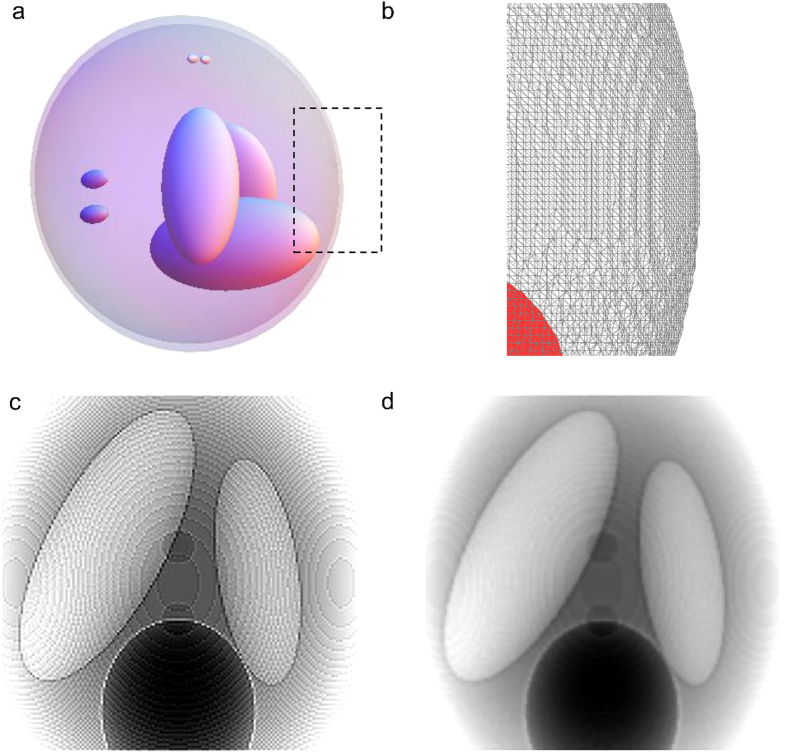
Discretization artifact in XPCI simulation due to voxelized phantom (Reproduced with permission from Ref. 33). (**a**) 3-D Shepp-Logan phantom; (**b**) zoom-in view of (**a**); (**c**) simulated XPCI image using (**a**); (**d**) simulated XPCI image using (**a**) after applying a Gaussian filter (full-width-at-half-maximum, 10 pixels).
